# Selective Types of Autophagy

**DOI:** 10.1155/2012/156272

**Published:** 2012-08-21

**Authors:** Fulvio Reggiori, Masaaki Komatsu, Kim Finley, Anne Simonsen

**Affiliations:** ^1^Department of Cell Biology and Institute of Biomembranes, University Medical Center Utrecht, 3584 CX, Utrecht, The Netherlands; ^2^Protein Metabolism Project, Tokyo Metropolitan Institute of Medical Science, Tokyo 156-8506, Japan; ^3^BioScience Center, San Diego State University, San Diego, CA 92182, USA; ^4^Department of Biochemistry, Institute of Basic Medical Sciences, University of Oslo, 0317 Oslo, Norway

The focus of this special issue of the International Journal of Cell Biology is to underscore the recent developments in the field of macroautophagy and how this degradative pathway intersects with cellular metabolism, complex physiological functions, and human diseases. During the last decade, autophagy has become an expanding field in biomedical life sciences due to its involvement with numerous intracellular processes. Autophagy also plays a role in pathology, and it has the therapeutic potential to be the target for the treatment of specific human diseases. Early studies suggested that autophagy was a nonselective process in which cytoplasmic structures were randomly sequestered into autophagosomes before being delivered to the mammalian lysosome or the plant and yeast vacuole for degradation. Now there is growing evidence that unwanted cellular structures can be selectively recognized and exclusively eliminated within cells (F. Reggiori et al., “*Selective types of autophagy*”). This is achieved through the action of specific autophagy receptors, as reviewed by C. Behrends and S. Fulda in “*Receptor proteins in selective autophagy*”) and studied by K. Marchbank et al. “*MAP1B interaction with the FW domain of the autophagic receptor Nbr1 facilitates its association to the microtubule network*”. Thus excess or damaged organelles including mitochondria (A. May et al., “*The many faces of mitochondrial autophagy: making sense of contrasting observations in recent research*”; Y. Hirota et al., “*The physiological role of mitophagy: new insights into phosphorylation events*”), peroxisomes (A. Till et al., “*Pexophagy: the selective degradation of peroxisomes*”), lipid droplets (R. Singh and A. Cuervo, “*Lipophagy: connecting autophagy and lipid metabolism*”), endoplasmic reticulum and ribosomes (E. Cebollero et al., “*Reticulophagy and ribophagy: regulated degradation of protein production factories*”) can be specifically sequestered by autophagosomes and targeted to the lysosome for degradation.

Importantly, there is growing evidence that selective autophagy subtypes also have a wide range of physiological functions. In yeast, the cytosol-to-vacuole (Cvt) pathway transports hydrolases into the vacuole, which is reviewed by M. Umekawa and D. Klionsky in “*The cytoplasm-to-vacuole targeting pathway: a historical perspective*”. In eukaryotes, autophagy plays a central role in both innate and acquired immunity. Further sequestration and elimination of invading pathogens such as *Salmonella* and *Staphylococcus aureus* have been exploited to study autophagosome biogenesis (T. Noda et al., “*Three-axis model for Atg recruitment in autophagy against Salmonella*”; M. Mauthe et al., “*WIPI-1 positive autophagosome-like vesicles entrap pathogenic Staphylococcus aureus for lysosomal degradation*”). In pancreas cells, autophagy has recently been shown to specifically turn over secretory granules, as described by M. Vaccaro in “*Zymophagy: selective autophagy of secretory granules*”. Dysregulation of autophagic function has been implicated in a growing list of disease processes and has underscored the selective or substrate-specific versions of the pathway. Examples in this special issue include the clearance of aggregates associated with neurological diseases, as reviewed by T. Lamark and T. Johansen in “*Aggrephagy: selective disposal of protein aggregates by macroautophagy*” and by I. Nezis in “*Selective autophagy in Drosophila*”. In terms of cancer biology, autophagy has been viewed as having dual roles in both tumor suppression and progression. K. Hughson et al. in “*Implications of therapy-induced selective autophagy on tumor metabolism and survival*” review how activation of autophagy selective forms can be used as a potential therapeutic approach for the treatment of specific cancers. Adding to the complexity of autophagic function and regulation, the article by K. Juenemann and E. Reits “*Alternative macroautophagic pathways*” explores alternative macroautophagic pathways that are independent of key core autophagy components such as Beclin-1 or Atg5. We expect future research on the mechanism and regulation of selective autophagy, and the physiological importance of this pathway in human disease will be very exciting and expand on the findings highlighted in this issue of IJCB. 

*Fulvio Reggiori* *Fulvio Reggiori* *Maasaki Komatsu* *Maasaki Komatsu* *Kim Finley* *Kim Finley* *Anne Simonsen* *Anne Simonsen*

